# Control of allergic rhinitis and asthma test – a formal approach to the development of a measuring tool

**DOI:** 10.1186/1465-9921-10-52

**Published:** 2009-06-17

**Authors:** Luis Nogueira-Silva, Sonia V Martins, Ricardo Cruz-Correia, Luis F Azevedo, Mario Morais-Almeida, António Bugalho-Almeida, Marianela Vaz, Altamiro Costa-Pereira, Joao A Fonseca

**Affiliations:** 1 Serviço de Biostatística e Informática Médica, Faculdade de Medicina da Universidade do Porto, Porto, Portugal; 2 Serviço de Biostatística e Informática Médica and CINTESIS – Centro de Investigação em Tecnologias e Sistemas de Informação em Saúde, Faculdade de Medicina da Universidade do Porto, Porto, Portugal; 3 Clínica Universitária de Pneumologia, Faculdade de Medicina da Universidade de Lisboa and Unidade de Imunoalergologia, Hospital CUF-Descobertas, Lisboa, Portugal; 4 Clínica Universitária de Pneumologia, Faculdade de Medicina da Universidade de Lisboa and Centro Hospitalar Lisboa Norte, Lisboa, Portugal; 5 Associação Portuguesa de Asmáticos, Porto, Portugal; 6 Serviço de Biostatística e Informática Médica, CINTESIS – Centro de Investigação em Tecnologias e Sistemas de Informação em Saúde and Faculdade de Medicina da Universidade do Porto and Serviço de Imunoalergologia, Hospital de S. João, EPE, Porto, Portugal

## Abstract

**Background:**

The concurrent management of allergic rhinitis and asthma (ARA) has been recommended by Allergic Rhinitis and its Impact on Asthma (ARIA) guidelines. However, a tool capable of assessing simultaneously the control of upper and lower airways diseases is lacking.

**Aim:**

To describe the studies conducted to design the control of ARA test (CARAT) questionnaire.

**Methods:**

We performed a literature review to generate a list of potentially important items for the assessment of control of ARA. A formal consensus development process, that used an innovative web-based application, was designed – 111 experts in ARA and 60 patients participated. At the final consensus meeting, 25 primary and secondary care physicians formulated the questions and response options. A qualitative feasibility study (n = 31 patients) was conducted to evaluate the comprehensibility of the questionnaire while testing two different designs.

**Results:**

Thirty-four potentially important items were identified. All the steps of the consensus process were completed in 2.5 months. The opinions of experts and patients lead to the formulation of 17 questions. At the feasibility study the instructions and wording problems were corrected and a semi-tabular format was chosen.

**Conclusion:**

A tool to measure the control of allergic rhinitis and asthma was developed using a comprehensive set of methodological steps ensuring the design quality and the face and content validity. Additional validation studies to assess the psychometric properties of the questionnaire have started.

## Introduction

Allergic rhinitis and asthma (ARA) are inflammatory diseases and are often associated. The lack of control of these diseases is responsible for a significant loss in patient's quality of life and an important socioeconomic burden [1,2]. According to international guidelines, achievement of disease control is the primary goal for the treatment of allergic respiratory diseases [1,3]. Allergic Rhinitis and its Impact on Asthma (ARIA) [1] guidelines have recently emphasized in the importance of a combined approach for both evaluation and management of ARA. Thus, measurement of disease control should consider, concurrently, the pathologies of upper and lower airways [4].

Based on the definition of asthma control of the Global Initiative for Asthma [3], chronic disease control can be characterized as patients experiencing minimal symptoms, having no limitations in their activities, having minimal requirement for rescue medications, having near normal physiological function, and experiencing infrequent exacerbations.

In addition to quality of life questionnaires, symptoms and severity scores [5-9], several questionnaires for assessing control have been developed for asthma in the last 10 years [10-13]. To a lesser extent the same has happened for allergic rhinitis [14-16] and control questionnaires are being validated [1]. However, no questionnaire for measuring the control of ARA concurrently has been developed. In fact, only Rhinasthma [15] assesses ARA concurrently, but it is designed for evaluation of health-related quality of life impairment.

The "Control of Allergic Rhinitis and Asthma Test" (CARAT) project [17] aims to develop a brief self-administered tool to quantify the degree of control of ARA in adult patients with a previous medical diagnosis of ARA.

A fundamental phase for the accuracy and usefulness of such a tool is its early development. A recent systematic review recommended that these early steps should include different methods to ensure the quality of the questionnaire's design [18].

In this article, we aimed to describe the studies conducted to design the Control of Allergic Rhinitis and Asthma Test questionnaire, namely an item generation process, a formal web-supported consensus process and a qualitative feasibility study.

## Questionnaire development

Conceptually, this tool was developed as a self-administered questionnaire to quantify the degree of control in adult patients with a previous diagnosis of ARA, to be applicable both in clinical practice and research settings. Therefore, it should be short and easy to complete [19], while being suitable to assess both individual variations and discriminate between groups of patients with different levels of control.

The questionnaire development comprised 3 fundamental steps: I) an item generation process, II) a formal consensus process and III) a feasibility study. These steps are described and discussed independently below.

## Item generation process

### Methods

**Figure 1:**
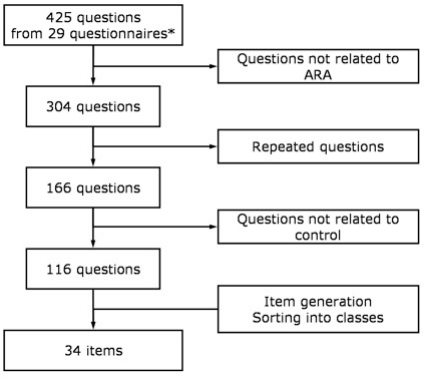
**Item generation flowchart**. From the questions retrieved from published questionnaires, the potentially important items were identified. *four were asthma control questionnaires; none were allergic rhinitis control questionnaires; 6 assessed ARA concurrently but none its control.

We performed a literature search in the Scopus database (which includes Medline and EMBASE) [20] with the following keywords: 'asthma', 'rhinitis', 'conjunctivitis', 'questionnaire', 'symptom score', 'control' and 'quality of life' which retrieved a total of 2693 articles. Additional questionnaires were retrieved from papers' references and from studies known to the authors. All the questionnaires were classified according to their purposes and diseases assessed. All their questions were tabulated. The list with all the questions was progressively reduced by first eliminating the questions not related to ARA, then the questions which were repeated or had similar meaning and finally those not related to control. The remaining questions were sorted into 10 classes (Lower airways symptoms, Nasal symptoms, Ocular symptoms, Oropharyngeal symptoms, Other symptoms, Activities, Sleep impairment, Psychosocial impact, Treatment and Exacerbation). This process allowed us to generate a list of potentially important items for the assessment of control of ARA (figure 1).

### Results

**Figure 2:**
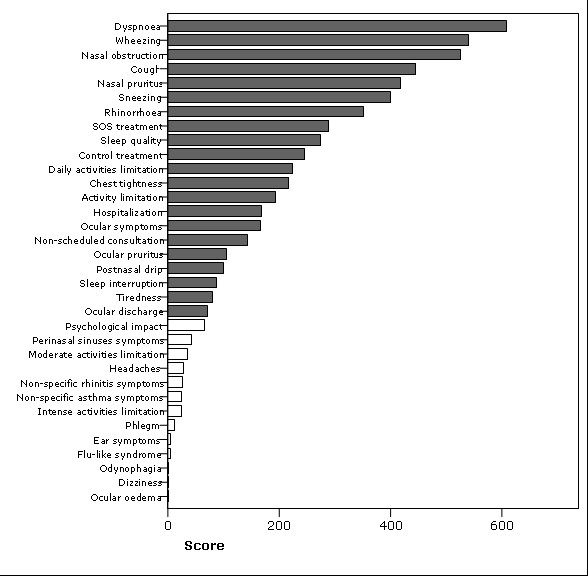
**Sorted items**. The 34 items from the item generation phase are presented, ranked by their score (inverse of the proportion of participants who chose the item, multiplied by the sum of its rankings). The shortlist (filled bars) included the highest scored items and the only item suggested by the participants ("Work/school absenteeism").

A total of 29 different questionnaires were identified. Only 4 questionnaires assessed disease control and were related to asthma control. The 29 questionnaires comprised 425 questions from which 116 were selected (figure 1). Thirty-four individual potentially important items were identified from the questions (figure 2) and were used in the consensus process.

### Discussion

The item generation derived from the comprehensive review of the existing literature, unlike the development of asthma control questionnaires [11-13].

It led to the creation of a list with all aspects related to the control of ARA. Moreover, both experts and patients were asked to suggest additional items in the following rounds.

## Formal consensus process

### Methods

A formal consensus development process was designed, informed by the Nominal Group Technique model modified by the RAND Corporation [21]. It comprised 1) a public presentation of the project at a national conference, 2) two rounds of experts' contribution via a web application with feedback after each round, 3) a round of patients' contribution and 4) a final consensus conference.

### Initial meeting

The project's objectives and outline were presented at the 2007 national annual meeting of the Portuguese Allergology and Clinical Immunology Society within a session that discussed the control of ARA. The audience was invited to answer questions related to the importance of a single questionnaire assessing simultaneously the control of allergic rhinitis and asthma, its appropriateness, usefulness and possible designs, using an on-site televoting system with immediate feedback. The audience responses are presented as proportions of the respondents for each question; between 28 and 55 delegates answered the different questions.

### Experts' contributions

Portuguese experts in ARA were invited to participate in the consensus process by email. The email addresses (n = 276) were provided by national scientific societies (Portuguese Allergology and Clinical Immunology Society and the Portuguese Pneumology Society) and other organized groups with interest in ARA.

A total of 111 (40%) experts participated: 81 participated in the first round and 82 in the second. Fifty-two experts participated in both rounds.

A web-based application was developed to allow online interaction with the experts. It gathered theirs input, provided them feedback at the end of each round, facilitated the data gathering and analysis, while maintaining anonymity of the answers (for further details see Additional file 1).

In the first round, each participant was asked to choose 15 items, either from the list of items generated previously or by suggesting new ones. Then, he/she was asked to rank those items by importance. Finally, for each of the chosen items, the participant had to choose between different characteristics of the items such as intensity or frequency.

For each item, a weighted score was calculated as the inverse of the proportion of participants who had chosen it, multiplied by the sum of its rankings. The items were ranked by this score and plotted in a bar graph.

A shortlist was elaborated with the items with the highest scores. This shortlist had to include at least two specific items from each class. In addition, the new items suggested by the participants were added to the shortlist.

In the second round, the participants were presented the shortlist of items generated at the end of the first round, grouped by classes, and were asked to rank the items within their classes. For each item we calculated the inverse of its average ranking, and multiplied it by the number of items in its class.

In addition, the participants were asked to choose the type of response options for each item between Visual Analogue Scales (VAS), different Likert scales or dichotomic scale. Standard descriptive statistical techniques were used.

### Patients' contribution

Sixty patients with ARA were enrolled at a hospital-based allergy outpatient clinic. Patients older than 18 years of age, with a medical diagnosis of ARA, able to read and fill the questionnaire and without other respiratory conditions were eligible for participation. Fifty-four were women, the average (standard deviation) age was 35 (14.4) years. Seventeen (29%) patients had four years of school education, 20 (35%) had less than twelve and 21 (36%) had twelve or more years of school education; 2 patients didn't provide this information.

The data was collected by a psychologist trained in questionnaire development. The patients were presented a paper form with the shortlist of items generated at the end of the experts' first round, grouped by classes. They were invited to suggest new items. Then, as in the experts' second round, the patients were asked to rank the items within their classes. For each item we calculated the inverse of its average ranking, multiplied it by the number of items in its class and summarized it in a table.

### Final meeting

Twenty-five Allergologists, Pneumologists and General Practitioners, with a special interest in ARA, from different regions of Portugal, were invited to participate in the final consensus meeting, independently of having or not participated previously in the project.

The session started with an oral presentation of the results of the previous rounds.

Subsequently, the participants were divided in 3 groups. Each group was attributed around 12 items belonging to 2 or 3 classes. The group was asked to discuss the results from the previous rounds and formulate a question and the response options for each item attributed to them. The authors provided the participants with examples of questions from existing questionnaires.

All participants were individually asked to score each item from 1 to 9 according to its importance (1 being the lowest). This data was processed on-site, presented to the panel and used to stimulate the discussion.

Finally, the panel was reassembled and discussed all the questions, focusing on different aspects related to the items and their importance, the wording of the questions, the response options and the timeframe to be assessed. At the end of the discussion, consensus was reached for all questions.

The meeting lasted approximately 7 hours.

## Results

### Initial meeting

At the initial public presentation, four fifths of the audience (81%) considered that questionnaires assessing the control of ARA would be useful for the clinical practice. Moreover, four fifths (81%) preferred a single questionnaire assessing ARA concurrently rather than 2 separate questionnaires. Nearly half (48%) considered 4 weeks to be the best time-period to be assessed in the questions. Nearly half (47%) considered that the questions should assess the frequency rather than the intensity of the symptoms while around one third (38%) considered that the option between frequency and intensity should be made for each individual question. More than half (59%) considered the best scale for the response options to be a Likert scale (with 4 points (26%), 5 points (20%) or 3 points (13%)), nearly one third (32%) preferred a VAS, while 9% preferred a dichotomic (Yes/No) scale.

### Experts' and patients' contribution

In the first round each expert chose 15 items and sorted them by order of importance. Nine out of the 81 participants (11%) only chose 15 items and didn't sort them by importance. The score calculated for each item is presented in figure 2.

After the first round, the shortlist had 22 items, including the only item suggested by the participants – "Work/school absenteeism".

Globally, for the 22 items, the participants preferred frequency rather than intensity.

The experts, in their second round, and the patients were asked to rank the 22 items within their classes. The comparison of the ranking by the 2 groups can be seen in table 1. Some differences occurred, mainly in the Nasal symptoms class and the Activities class. The greatest difference occurred with the item "Tiredness".

**Table 1:** Items ranked within classes by the experts (n = 82) at the second round and by patients (n = 60).

	**Experts**	**Patients**
**Lower airways symptoms**		
Dyspnoea	**2,62**	**2,22 (1^st^)**
Wheezing	1,80	1,95 (2^nd^)
Cough	1,35	1,41 (4^th^)
Chest tightness	1,21	1,46 (3^rd^)
**Nasal symptoms**		
Nasal obstruction	**2,81**	1,84 (2^nd^)
Sneezing	1,80	**1,95 (1^st^)**
Rhinorrhoea	1,67	1,55 (4^th^)
Nasal pruritus	1,58	1,84 (2^nd^)
Postnasal drip	1,17	1,32 (5^>th^)
**Ocular symptoms**		
Ocular pruritus	**2,02**	**2,17 (1^st^)**
Ocular symptoms	1,40	1,2 (3^rd^)
Ocular discharge	1,27	1,42 (2^nd^)
**Activities**		
Daily activities limitation	**2,45**	1,80 (2^nd^)
Activity limitation	1,80	1,47 (3^rd^)
Work/school absenteeism	1,31	1,13 (4^th^)
Tiredness	1,30	**2,64 (1^st^)**
**Sleep impairment**		
Sleep interruption	**1,37**	**1,41 (1^st^)**
Sleep quality	1,30	1,26 (2^nd^)
**Treatment**		
Control treatment	**1,59**	**1,67 (1^st^)**
SOS treatment	1,15	1,11 (2^nd^)
**Exacerbation**		
Non-scheduled consultation	**1,41**	**1,36 (1^st^)**
Hospitalization	1,26	1,30 (2^nd^)

The items are shown ranked according to the inverse of their average ranking by the participants, multiplied by the number of items in the class. In bold are the items with highest score and in brackets are indicated the order of the ranking by patients. Differences between experts and patients are seen in the Nasal symptoms class ("Sneezing" and "Nasal obstruction") and the Activities class ("Tiredness").

As for the type of response options, the experts preferred a Likert scale for 16 items while a dichotomic (Yes/No) scale was preferred for the remaining 6 items ("Work/school absenteeism", "Postnasal drip", "Ocular symptoms", "Control treatment", "Non-scheduled consultation" and "Hospitalization"). The VAS wasn't preferred for any of the items; in fact it was the least chosen option and its highest proportion of choice was 37%, for the item "Nasal obstruction".

### Final meeting

Within three groups, the physicians discussed and formulated the questions and response options for the items distributed to each group.

The discussion led to the formulation of 17 questions. The item "Postnasal drip", which had a low ranking, was substituted by a more general item "Throat complaints, like pruritus or postnasal drip". The 3 items regarding Ocular symptoms, generally low ranked, were aggregated in a single question. The panel considered the items "Activity limitation", "Daily activities limitation" and "Tiredness" redundant and rephrased these items. The possibility of including "Sports activities absenteeism" in the question referring to the item "Work/school absenteeism" was discussed. The item "Sleep quality" was considered to have different interpretations and was replaced by "Symptoms/complaints after awaking". The items "Control treatment" and "SOS treatment" were aggregated in one single question as "Increase in the use of medication".

The panel decided that 4 weeks should be the timeframe used for every question. It was considered a period long enough to have a perspective on control without great distortion of the patients recollection of events.

When applicable, all the questions referred to frequency of occurrence, rather than intensity. The response options for the questions related to Lower airways symptoms, Nasal symptoms, Ocular symptoms, Sleep impairment and Activities (apart from "Work/school/sports activities absenteeism") should be 4-points Likert scales. For each class the response options were formulated according to the existing guidelines. For the remaining questions, a dichotomic (Yes/No) scale was used.

The participants agreed to test, in the following feasibility study, two versions of the questionnaire, one with a question-answer format and another with a tabular format.

The whole formal consensus process was completed in 2.5 months.

## Discussion

A formal consensus process was designed to reduce the number of items, determining which should be included in the questionnaire. To do so, it was considered necessary to obtain the opinions of experts in ARA but also to have a contribution from patients with ARA, unlike other studies which only include patients in the feasibility or validation studies [11-13].

The communication with the experts was considerably simplified by the use of an innovative web-based platform. This platform allowed to contact a large number of experts in ARA, made the data collection and processing more efficient while maintaining the safety and anonymity of the information and allowed to provide the participants with feedback on the rounds' results.

In this way, the contribution of ARA experts and patients through several steps with different methodological approaches, allowed the identification of 22 important items to assess the control of these diseases. The importance given to the items progressively converged. Discrepancies occurred in a few items, particularly in the "Activities" and "Nasal symptoms" classes. These discrepancies may result from different interpretations of the concepts to which the items refer to. For instance, during the consensus meeting, experts considered "Activities limitation" to be equivalent to what patients considered "Tiredness". Moreover, the gender imbalance in the patients sample may have contributed for these differences.

The final meeting allowed for a thorough discussion of the items and formulation of the questions and the response options for the questionnaire by primary and secondary care physicians.

ARIA proposes VAS to be used in the evaluation of rhinitis severity [1]. This approach has been successfully tested in recent studies. However, both at the initial meeting and at the consensus rounds, the experts preferred either Likert scales or dichotomic (Yes/No) scales as the response options for all the items.

## Feasibility study

### Methods

Thirty-one patients with ARA were enrolled at a hospital-based allergy outpatient clinic. Patients older than 18 years of age, with a medical diagnosis of ARA, able to read and fill the questionnaire and without other respiratory conditions were eligible for participation. Twenty-eight were women, the average (standard deviation) age was 32 (14.6) years. Six (19%) patients had four years of school education, 12 (39%) had less than twelve and 13 (42%) had twelve or more years of school education.

The data was collected by a psychologist trained in questionnaire development.

The patients were presented two versions of the questionnaire, different in their format. In the first version each question was followed by its response options (question-answer format). In the second version, the questions and response options were presented as tables (tabular format). Both versions started with a short sentence with the filling instructions.

All patients completed both versions of the questionnaire, alternately starting with either the tabular format version or the question-answer format version. Sixteen patients completed the tabular format questionnaire first. The time they took to complete each version was measured. The difficulties each patient had while completing each version were registered, as well as their opinions regarding the wording and comprehensibility of the questions. The preferred version and the reasons for the choice were also enquired. The data was processed with standard descriptive statistic techniques.

### Results

Overall the patients presented few difficulties completing either version. Two patients needed assistance to fill the questionnaire.

Regarding the tabular format, 19 patients didn't report any difficulties. Seventeen comments were registered, 9 of them regarding the instructions of the questionnaire and 6 related to the wording of the questions.

As for the question-answer format, 21 patients didn't report any difficulties filling the questionnaire. Thirteen comments were registered, 5 of them concerning the wording of the questions and 5 related to the response options.

Twenty-three patients (74%) preferred the tabular format and all stated this version was easier and quicker to fill. All those who preferred the question-answer format considered it to be easier to understand, with more detailed information.

The patients took on average (standard deviation) 3' (3.1) to fill the tabular format version (minimum 1', maximum 12') and 5' (4.9) to fill the question-answer format version (minimum 2', maximum 20'). The patients took on average (standard deviation) 1.9' (2.29) less to fill the tabular version of the questionnaire.

### Discussion

The comments of the patients were used to construct a final version of the questionnaire, correcting important issues. In the final version, greater emphasis was given to the instructions of the questionnaire, since the disregard of these directives was one of the biggest problems found. The wording of some questions was altered to increase comprehensibility. The wording of the response options was changed as it was judged preferable to maintain the same options throughout the questionnaire.

The test of two different formats of the questionnaire showed that the tabular format was preferred by most patients and was quicker to complete. These results weren't influenced by the order of administration of the 2 versions of the questionnaire (data not shown). However, 26% of the patients considered the question-answer format to be easier to understand. The chosen layout for the questionnaire was a semi-tabular format, in order to make it as clear as possible to the patients.

## Conclusion

We describe a formal methodological approach for the development of a tool to concurrently measure the control of allergic rhinitis and asthma, in previously diagnosed adult patients.

Questionnaires for the assessment of control of either asthma or rhinitis are in use or under development. However, a tool capable of assessing simultaneously the control of upper and lower airways diseases was lacking.

The questionnaire now developed achieves this purpose (the direct English translation from Portuguese version is available in submitted Additional file 2). However, it has 17 questions, a considerable number that may hinder its use in some settings. Additional studies to evaluate the questionnaire's psychometric properties, reduce the number of items through analytical processes, establish the scoring system and assess its usefulness in clinical practice are being carried out.

Moreover, we propose a questionnaire development approach with a sequence of qualitative methods, including an innovative consensus process, supported by web technologies. It was designed to allow for a large participation of stakeholders, drawing together the opinions of experts and patients.

This proposal is in agreement with recently published quality criteria for questionnaire development [18] and its different procedures were carried out in order to ensure the face and content validity and the overall quality of the instrument's design.

## Abbreviations

ARA: allergic rhinitis and asthma; ARIA: Allergic Rhinitis and its Impact on Asthma; CARAT: Control of Allergic Rhinitis and Asthma Test; PHP: Hypertext Preprocessor; VAS: visual analogue scale.

## Competing interests

The authors declare that they have no competing interests.

## Authors' contributions

LNS participated in data collection, analysis and wrote the manuscript draft, SVM participated in data collection and analysis and reviewed the manuscript, RCC participated in study design, web application design, data analysis and reviewed the manuscript; LFA participated in study design, data analysis and reviewed the manuscript, MMA, ABM and MV participated in the study conception, led the consensus meeting and reviewed the manuscript, ACP participated in the study design and manuscript writing, JAF is responsible for the CARAT project and participated in all stages and tasks. All authors have read and approved the final manuscript.

## Supplementary Material

Additional file 1 **Description of the use of a web application in the consensus process for the development of the Control of Allergic Rhinitis and Asthma Test (CARAT)**. The data provide more details on the development and use of the web-based consensus application. Click here for file

Additional file 2 **CARAT – Control of Allergic Rhinitis and Asthma Test**. This is a translation to English of the of the preliminary version of Control of Allergic Rhinitis and Asthma Test, the development of which is described in this paper. Click here for file
