# Supplementary material for: Control of allergic rhinitis and asthma test – a formal approach to the development of a measuring tool
Source: Respir Res. 2009 Jun 17;10(1):52. doi: 10.1186/1465-9921-10-52 (PMC2706215; doi:10.1186/1465-9921-10-52)
Supplement: Additional file 1 — Description of the use of a web application in the consensus process for the development of the Control of Allergic Rhinitis and Asthma Test (CARAT). The data provide more details on the development and use of the web-based consensus application. [file 1465-9921-10-52-S1.doc]

**Additional file 1**

**Description of the use of a web application in the consensus process for the development of the Control of Allergic Rhinitis and Asthma Test (CARAT).**

A web-based application was developed to allow online interaction with the experts. The application is implemented as a component for a web content manager (Joomla! (1)), using PHP programming language (2) and a MySql (3) relational database management system.

Each e-mail invitation included a personalized link which granted direct access to this web application. The link contained a hash key generated by an md5 algorithm (4), avoiding the need for login. This prevented duplication of data entries by the same participant and unauthorized access to the application. This procedure allowed the investigators to know which participants had already completed each round while maintaining anonymity of the answers. A new e-mail was sent to all the experts at the beginning of the second round, with the same personalized link to the web application.

At each round, two e-mails reminding the experts who hadn’t participated were sent in the following weeks. Feedback on the results of the previous round was provided to the participants, both by e-mail and through the web application (figure 1).

1. LeBlanc J. Learning Joomla! 1.5 Extension Development: Creating Modules, Components, and Plugins with PHP: Packt Publishing; 2007.

2. PHP: Hypertext Preprocessor. Available from: [http://www.php.net](http://www.php.net/)

3. MySQL. Available from: [http://dev.mysql.com](http://dev.mysql.com/)

4. Rivest R. The MD5 Message-Digest Algorithm: MIT Laboratory for Computer Science and RSA Data Security, Inc.; 1992.


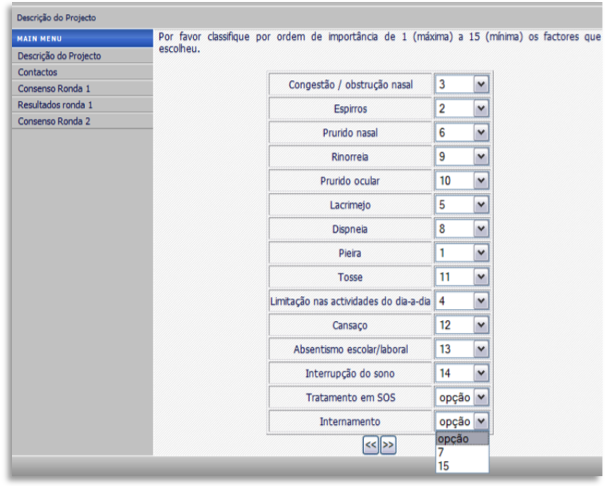

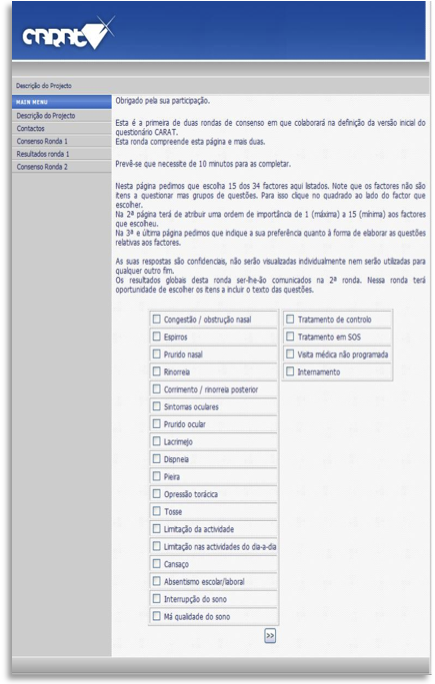
Figure 1 – Screenshots of the web application; a and b examples of data input; c and d feedback of results

a

b


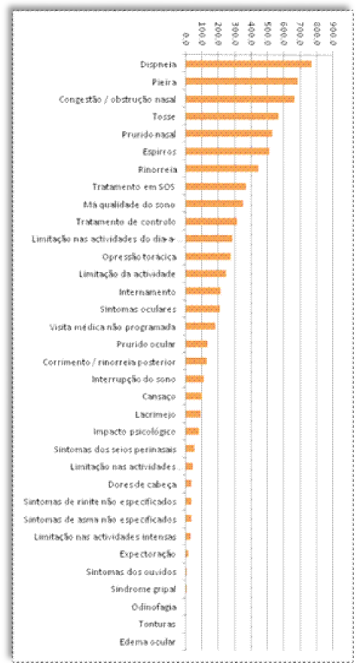

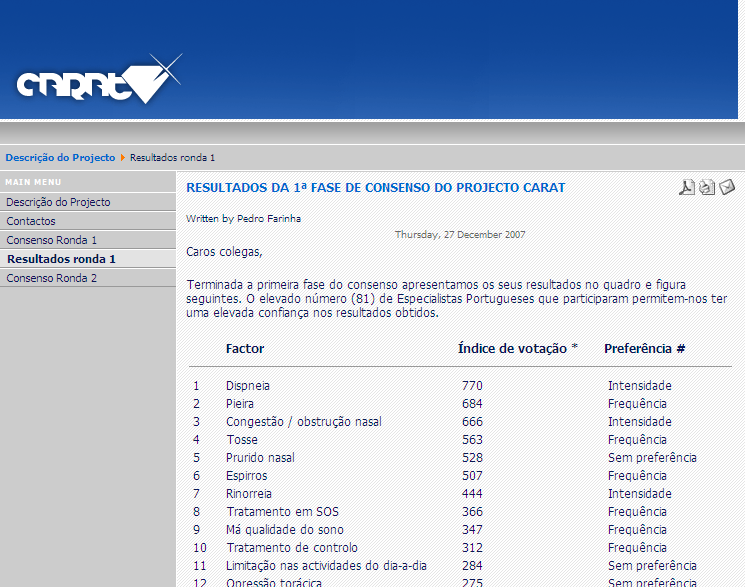


c

d
