# Supplementary material for: Control of allergic rhinitis and asthma test – a formal approach to the development of a measuring tool
Source: Respir Res. 2009 Jun 17;10(1):52. doi: 10.1186/1465-9921-10-52 (PMC2706215; doi:10.1186/1465-9921-10-52)
Supplement: Additional file 2 — CARAT – Control of Allergic Rhinitis and Asthma Test. This is a translation to English of the of the preliminary version of Control of Allergic Rhinitis and Asthma Test, the development of which is described in this paper. [file 1465-9921-10-52-S2.doc]

## Asthma and Allergic Rhinitis Control Test – CARAT (preliminary version)

*This questionnaire needs validation studies. Its scoring and psychometric properties are not currently known***.**

**Name:** ________________________________________________________________ **Date:** ____/____/____

**Gender:** Male 🞎 Female 🞎 Age**:** _______

Please choose the answer **☒** that **better describes** how you have felt **over the last 4 weeks**, due to your respiratory/allergic (asthma/rhinitis/allergy) disease.

During the last **4 weeks**, due to your **respiratory/allergic** (asthma/rhinitis/allergic) **disease**, how many times did you have:

|  | **Never** | **1 or 2 days**  **per week** | **More than 2**  **days per week** | **Almost everyday or every day** |
| --- | --- | --- | --- | --- |
| 1. **Nasal obstruction?** | 🞎 | 🞎 | 🞎 | 🞎 |
| 1. **Sneezes?** | 🞎 | 🞎 | 🞎 | 🞎 |
| 1. **Nasal itching?** | 🞎 | 🞎 | 🞎 | 🞎 |
| 1. **Nose dripping?** | 🞎 | 🞎 | 🞎 | 🞎 |
| 1. **Throat symptoms such as itching, tickling or a feeling of sputum in the throat?** | 🞎 | 🞎 | 🞎 | 🞎 |
| 1. **Eye symptoms such as itching, weeping or inflammation?** | 🞎 | 🞎 | 🞎 | 🞎 |

During the last **4 weeks**, due to your **respiratory/allergic** (asthma/rhinitis/allergic) **disease**, how many times did you have:

|  | **Never** | **1 or 2 days**  **per week** | **More than 2**  **days per week** | **Almost everyday or every day** |
| --- | --- | --- | --- | --- |
| 1. **Shortness of breath/dyspnoea?** | 🞎 | 🞎 | 🞎 | 🞎 |
| 1. **Wheezing?** | 🞎 | 🞎 | 🞎 | 🞎 |
| 1. **Chest tightness after physical efforts?** | 🞎 | 🞎 | 🞎 | 🞎 |
| 1. **Cough?** | 🞎 | 🞎 | 🞎 | 🞎 |

**Please continue to the next page**

|  | **Never** | **1 or 2 days**  **per week** | **More than 2**  **days per week** | **Almost everyday or every day** |
| --- | --- | --- | --- | --- |
| 1. During the the last **4 weeks**, due to your **respiratory/allergic** (asthma/rhinitis/allergic) **disease**, how many times did you feel **tired, with difficulty doing your daily activities/work?** | 🞎 | 🞎 | 🞎 | 🞎 |

During the last **4 weeks**, due to your **respiratory/allergic** (asthma/rhinitis/allergic) **disease**, how many times did you:

|  | **Never** | **1 or 2 days**  **per week** | **More than 2**  **days per week** | **Almost everyday or every day** |
| --- | --- | --- | --- | --- |
| 1. **Woke up during the night?** | 🞎 | 🞎 | 🞎 | 🞎 |
| 1. **Had complaints/symptoms in the morning, when you wake up?** | 🞎 | 🞎 | 🞎 | 🞎 |

|  | **Yes** | **No** | **Currently, I don't work/study** |
| --- | --- | --- | --- |
| 1. During the last **4 weeks**, due to your **respiratory/allergic** (asthma/rhinitis/allergic) **disease**, did you had to   **miss work/school?** | 🞎 | 🞎 | 🞎 |

|  | **Never** | **Less than**  **7 days** | **7 or more days** | **I'm not taking medication** |
| --- | --- | --- | --- | --- |
| 1. During the last **4 weeks**, due to your **respiratory/allergic** (asthma/rhinitis/allergic) **disease**, how many times did you have to **increase the use of medication?** | 🞎 | 🞎 | 🞎 | 🞎 |

During the last **4 weeks**, due to your **respiratory/allergic** (asthma/rhinitis/allergic) **disease,** did you need

|  | **Yes** | **No** |
| --- | --- | --- |
| 1. **To go to a doctor?** | 🞎 | 🞎 |
| 1. **To be hospitalized?** | 🞎 | 🞎 |

## *Thank you*
